# Beta-Blockers in Stable Coronary Artery Disease: A Systematic Review and Meta-Analysis of Observational Studies

**DOI:** 10.31083/RCM44520

**Published:** 2025-12-23

**Authors:** Jing-Xuan Liu, Shi-Yue Zheng, Fei Guo, Chun-Hui He, Jing Lin, Hao Fu, Xin Du, Jian-Zeng Dong

**Affiliations:** ^1^Department of Cardiology, Beijing Anzhen Hospital, Capital Medical University, 100029 Beijing, China

**Keywords:** beta-blockers, stable coronary artery disease, meta-analysis, cardiovascular outcomes, preserved left ventricular function

## Abstract

**Background:**

The efficacy of beta-blockers in stable coronary artery disease (CAD) patients with preserved left ventricular function remains controversial. We aimed to evaluate the cardiovascular associations of beta-blocker therapy in this population through a comprehensive meta-analysis.

**Methods:**

We conducted a systematic review and meta-analysis following Preferred Reporting Items for Systematic Reviews and Meta-Analyses (PRISMA) guidelines, searching PubMed, EMBASE, Web of Science, Scopus, Google Scholar, and Cochrane databases from inception to May 2025, updating and extending the previous meta-analysis. We included observational studies comparing beta-blocker therapy versus control in stable CAD patients, defined as those without acute coronary syndrome manifestations for a sufficient period (typically >6 months) to ensure clinical stability, with preserved left ventricular ejection fraction (left ventricular ejection fraction >50%). Primary outcome was cardiac death. Secondary outcomes included all-cause mortality, heart failure, myocardial infarction (MI), and stroke. Random-effects models were used for all analyses. Subgroup analyses were conducted for cardiac and all-cause death stratified by propensity score matching status and prior beta-blocker use exclusion criteria. Publication bias was assessed using funnel plots and Peter's test.

**Results:**

Nine observational studies encompassing 903,870 patients (616,645 beta-blocker users vs. 287,225 controls) were included. Beta-blocker therapy showed no significant association with the primary endpoint: cardiac death (hazard ratio (HR) 0.98, 95% CI: 0.93–1.04, *p =* 0.54). Secondary outcomes similarly demonstrated no significant associations: all-cause mortality (HR 0.98, 95% CI: 0.91–1.05, *p =* 0.49), MI (HR 1.02, 95% CI: 0.93–1.11, *p =* 0.72), stroke (HR 1.02, 95% CI: 0.97–1.08, *p =* 0.43), and heart failure (HR 1.10, 95% CI: 0.95–1.27, *p =* 0.20). Substantial heterogeneity was observed for all-cause death (I^2^ = 87%) and heart failure (I^2^ = 95%). Subgroup analyses failed to identify populations with clear associations between beta-blocker therapy and improved outcomes.

**Conclusion:**

Beta-blocker therapy was not significantly associated with cardiovascular benefits in stable CAD patients with preserved left ventricular function. These findings provide additional contemporary evidence supporting current guideline recommendations from both American Heart Association (AHA)/American College of Cardiology (ACC) and European Society of Cardiology (ESC) regarding beta-blocker use in this population. Clinicians should conduct individualized risk-benefit assessments rather than adopting routine prescribing patterns.

**The PROSPERO Registration:**

CRD420251141812, https://www.crd.york.ac.uk/PROSPERO/display_record.php?RecordID=1141812.

## 1. Introduction

Coronary artery disease (CAD) remains the leading cause of cardiovascular mortality worldwide, affecting millions of patients and significantly impacting their quality of life and prognosis [1]. Stable CAD, characterized by coronary artery stenosis resulting in myocardial ischemia while maintaining relatively stable clinical presentation without acute coronary syndrome manifestations, remains a significant clinical challenge [1, 2]. Despite remarkable progress in revascularization strategies and pharmacological interventions, patients with stable CAD continue to experience considerable risks of major adverse cardiovascular events (MACE), encompassing myocardial infarction (MI), stroke, and cardiovascular mortality [3]. Therefore, identifying effective pharmacological intervention strategies to improve long-term outcomes in this patient population holds substantial clinical significance.

Beta-blockers, as one of the cornerstones of cardiovascular pharmacotherapy, exert cardioprotective effects through blockade of β-adrenergic receptors [4]. Their primary mechanisms include reducing heart rate, decreasing myocardial contractility, and lowering blood pressure, thereby reducing myocardial oxygen consumption, increasing ischemic threshold, and potentially improving perfusion of ischemic areas through prolongation of diastole and redistribution of myocardial blood flow [5]. However, the universal benefit of beta-blockers in post-MI patients has been challenged by recent evidence. The REDUCE-AMI trial—a large randomized controlled study of 5020 patients with acute MI and preserved ejection fraction—found no reduction in the composite endpoint of death or recurrent MI with beta-blocker therapy over 3.5 years of follow-up [6]. Furthermore, a recent comprehensive meta-analysis by Chi *et al*. (PMID: 39298680) [7] demonstrated that in the contemporary reperfusion era, beta-blocker use post-MI in patients with preserved ejection fraction may not only fail to confer mortality benefits beyond a 1-year event-free period, but could also be associated with detrimental outcomes, including a significant increase in major adverse cardiac and cerebrovascular events (hazard ratio (HR) 1.24; 95% CI: 1.01–1.52). Notably, the evidence for beta-blockers in stable CAD without prior MI or left ventricular dysfunction is even more limited and contentious. While their theoretical benefits (e.g., reduced myocardial oxygen demand) are well-established, clinical evidence supporting routine use in this population remains largely extrapolated from post-MI studies, lacking direct validation from rigorous trials.

The most comprehensive evaluation to date was a 2021 meta-analysis by Arero *et al*. [8], which pooled six observational studies (n = 774,089) and found no significant reduction in MACE MI, or cardiovascular mortality with beta-blocker therapy. These results questioned the widespread use of beta-blockers in stable CAD and underscored a critical gap between clinical practice and evidence-based recommendations. However, in 2023, Godoy *et al*. [9] published a large population-based cohort study in the Journal of the American College of Cardiology that challenged these findings. Using a rigorous new-user design in 28,039 patients with angiographically confirmed stable CAD, they demonstrated an 8% relative risk reduction in the composite of all-cause death and hospitalization for heart failure (HF) or MI (HR 0.92, 95% CI: 0.86–0.98, *p* = 0.006). This contradiction in recent evidence highlighted the ongoing uncertainty regarding beta-blocker efficacy in this population. Therefore, this systematic review and meta-analysis aims to update and expand upon the existing evidence by integrating all available studies to clarify the role of beta-blockers in stable CAD and guide contemporary therapeutic decision-making.

## 2. Methods

### 2.1 Protocol and Literature Search Strategy

This systematic review and meta-analysis was conducted in accordance with the Preferred Reporting Items for Systematic Reviews and Meta-Analyses (PRISMA) statement and the Meta-analysis of Observational Studies in Epidemiology (MOOSE) guidelines. https://www.crd.york.ac.uk/PROSPERO/display_record.php?RecordID=1141812 (or equivalent view link for CRD420251141812).

We conducted a systematic literature search of PubMed, EMBASE, Web of Science, Scopus, Google Scholar, and Cochrane Controlled Trials Register from database inception to May 24, 2025, to identify studies evaluating the effects of beta-blockers on cardiovascular outcomes in patients with stable CAD. Search terms included “beta-blockers”, “beta blockers”, “beta antagonists”, “adrenergic beta-antagonists”, “stable coronary artery disease”, “stable CAD”, “ischemic heart disease”, “major adverse cardiovascular events”, “MACE”, “cardiovascular death”, “myocardial infarction”, and “stroke”.

### 2.2 Study Selection, Eligibility Criteria, and Outcome Measures

For the purpose of this meta-analysis, stable CAD was defined as coronary artery stenosis without acute coronary syndrome manifestations for a sufficient period to ensure clinical stability. This included patients with significant coronary stenosis without prior MI, patients stabilized after elective Percutaneous Coronary Intervention (PCI) or CABG (generally >6 months post-procedure), and patients with remote MI without recurrent events (typically >6 months since the last event). Left ventricular dysfunction was defined as left ventricular ejection fraction (left ventricular ejection fraction [LVEF]) ≤50%.

The inclusion criteria were as follows: (1) studies involving patients with stable CAD or stable ischemic heart disease as defined above; (2) comparison of beta-blocker therapy versus control (placebo or no beta-blocker treatment); (3) reporting of at least one cardiovascular outcome of interest (all-cause death, cardiac death, HF, MI, or stroke); (4) Follow-up duration of at least 12 months. The exclusion criteria were as follows: (1) studies focusing on patients with AMI or left ventricular dysfunction; (2) animal studies; (3) case reports, editorials, comments, reviews, and meta-analyses; (4) studies published in languages other than English; (5) conference abstracts without full-text availability; (6) studies with insufficient data for analysis. The primary outcome was cardiac death. Secondary outcomes included all-cause death, HF, MI, and stroke.

Two investigators independently screened all retrieved records by title and abstract, followed by full-text review of potentially eligible studies. Disagreements were resolved through discussion with a third reviewer.

### 2.3 Data Extraction and Quality Assessment

Data extraction was performed independently by two reviewers using a standardized data extraction form. The following information was extracted: study characteristics (first author, publication year, study design, location, sample size, follow-up duration), patient demographics (age, sex, body mass index, diabetes, hypertension, hypercholesterolemia, stroke history, smoking status), procedural characteristics (percutaneous coronary intervention, coronary artery bypass grafting), concomitant medications (statins, aspirin, angiotensin converting enzyme (ACE) inhibitors/angiotensin receptor blockers, calcium channel blockers), confounding control methods (propensity score matching or multivariate adjustment), and outcome data (risk ratios or hazard ratios with 95% confidence intervals for each cardiovascular outcome).

The methodological quality and risk of bias of included studies were assessed using the Risk Of Bias In Non-randomized Studies - of Interventions (ROBINS-I) tool.

### 2.4 Statistical Analysis

All analyses were performed using R statistical software (version 4.3.0, R Foundation for Statistical Computing, Vienna, Austria) with the meta package and RevMan (version 5.4). HR with 95% confidence intervals (CI) were calculated for all outcomes. Statistical heterogeneity between studies was assessed using the I^2^ statistic and Cochran’s Q test. According to Higgins *et al*. [10], I^2^ values of <25%, 25–49%, 50–75%, and >75% represent no, low, moderate, and high levels of heterogeneity, respectively.

Random-effects models (using the DerSimonian-Laird method with REML tau^2^ estimation) were applied for all analyses. Additionally, due to the relatively small number of included studies (<10), the Hartung-Knapp adjustment was applied to provide more conservative estimates. Publication bias was assessed using visual inspection of funnel plots and Peter’s regression test. Subgroup analyses were conducted for cardiac and all-cause death stratified by propensity score matching status and prior beta-blocker use history. All subgroup analyses were performed using random-effects models.

## 3. Results

### 3.1 Study Selection and Publication Bias Assessment

**Fig. 1.**
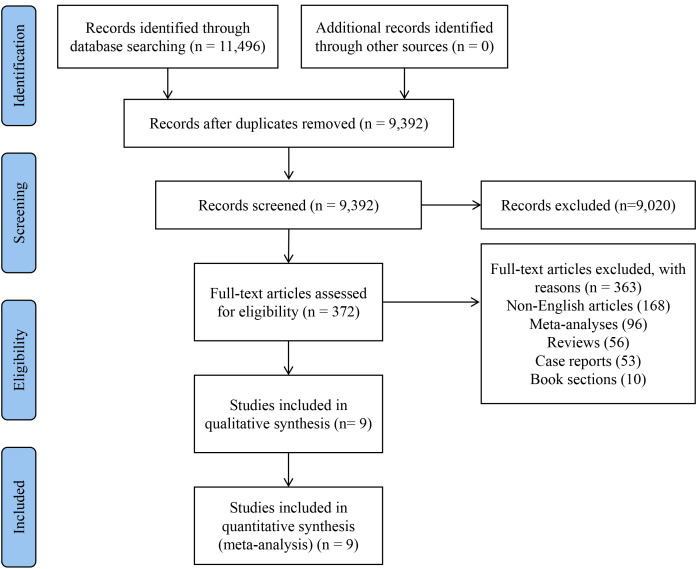
**Flow diagram of meta-analysis**.

We used the Preferred Reporting Items for Systematic Reviews and Meta-Analyses flow chart to describe the screening and selection of articles (Fig. 1). After a comprehensive search, we identified 11,496 potentially relevant articles from database searching. After removing 2104 duplicates, 9392 records were screened by title and abstract, and 372 studies were selected for full-text review. After applying the inclusion and exclusion criteria, 9 studies published between 2005 and 2025 were finally analyzed [9, 10, 11, 12, 13, 14, 15, 16, 17].

The 9 studies comprised 903,870 patients with stable CAD, of whom 616,645 patients received beta-blocker therapy and 287,225 patients served as controls. The baseline characteristics of the included studies are presented in Table 1 (Ref. [9, 11, 12, 13, 14, 15, 16, 17, 18]), and the baseline characteristics of the patients are shown in Table 2 (Ref. [9, 11, 12, 13, 14, 15, 16, 17]). The studies were conducted across various locations including the United States, international multi-center settings, Taiwan, Japan, South Korea, and Canada. Six studies were retrospective in design, while 3 were prospective. The median follow-up duration ranged from 3 to 5.4 years. Propensity score matching was employed in 6 studies, while 3 studies used multivariate adjustment to control for confounding factors. Only 2 studies explicitly excluded patients with prior beta-blocker use, while the remaining 7 studies did not specify or exclude patients with previous beta-blocker exposure.

**Table 1.** **Characteristics of included studies**.

Studies	Location	Study periods	Control for confounding	Timing of the study	Follow-up periods, median (year)	Without prior BB use	Total cohort	BB	no BB	Primary outcomes	Secondary outcomes
Bunch *et al*., 2005 [12]	Single center, USA	1993−2002	Multivariate	Prospective	3	No	4304	1024	3280	All-cause death	All-cause death, MI
Bangalore *et al*., 2012 [11]	Multi centers, International	2003–2009	PS matched	Prospective	3.6	No	7198	3599	3599	Cardiac death, nonfatal MI, or nonfatal stroke	Cardiac death, nonfatal MI, nonfatal stroke, hospitalization for atherothrombotic events, and revascularization
Li *et al*., 2013 [13]	Single center, Taiwan	1997–2003	Multivariate	Prospective	5.4	No	607	243	364	All-cause death, cardiac death, non-cardiac death	-
Ozasa *et al*., 2013 [15]	Multi centers, Japan	2005–2007	PS matched	Prospective	3	No	5288	1117	4171	Cardiac death, MI	All-cause death, cardiac death, MI, revascularization
Motivala *et al*., 2016 [14]	Multi centers, USA	2005–2013	Multivariate	Retrospective	3	No	755,215	539,521	215,694	All-cause death	Revascularization, hospitalization for MI, HF, or stroke
Tsujimoto *et al*., 2017 [16]	Multi centers, International	2001–2005	PS matched	Retrospective	5	No	1477	1019	458	All-cause death	All-cause death, MI or stroke
Lee *et al*., 2022 [17]	Multi centers, South Korea	2005–2015	PS matched	Retrospective	5	No	78,380	45,746	32,634	MACE: composite of cardiac death, MI, HF, and hospitalization for 5 years after PCI with 6 months quarantine	All-cause death and the individual MACE components
Godoy *et al*., 2023 [9]	Single center, Canada	2009–2019	PS matched	Retrospective	5.2	Yes	28,039	12,695	15,344	All-cause death and hospitalization for HF or MI	All-cause death and hospitalization for HF or MI, cardiac death, revascularization, hospitalization for stroke or unstable angina
Khan *et al*., 2025 [18]	Multi centers, USA	2009–2024	PS matched	Retrospective	5	Yes	23,362	11,681	11,681	All-cause death	Hospitalization for MI, stroke, HF, and AF

Abbreviation: BB, Beta-blocker; PS, production sequence; MI, myocardial infarction; HF, heart failure; MACE, major adverse cardiovascular events; AF, atrial fibrillation; PCI, Percutaneous Coronary Intervention.

**Table 2.** **Baseline patient characteristics**.

Studies	Total cohort	Age (years), mean	Men, (%)	BMI (kg/m^2^), mean	Diabetes, (%)	Hypertension, (%)	Hypercholesterolaemia, (%)	Stroke, (%)	Smoking, (%)	PCI, (%)	CABG (%)	Statins, (%)	Aspirin, (%)	ACEi/ARB, (%)	CCB, (%)
Bunch *et al*., 2005 [12]	4304	65	75	-	16	60	55	-	23	30	20	18	-	43	-
Bangalore *et al*., 2012 [11]	7198	69	66	28	39	81	73	12	9	-	-	71	74	44	42
Li *et al*., 2013 [13]	607	67	70	26	34	75	-	8	27	56	23	19	-	38	54
Ozasa *et al*., 2013 [15]	5288	68	72	24	37	83	-	10	25	-	-	52	99	46	56
Motivala *et al*., 2016 [14]	755,215	65	64	30	35	82	-	-	19	30	-	-	-	-	-
Tsujimoto *et al*., 2017 [16]	1477	62	69	-	100	83	81	-	11	-	-	72	87	-	-
Lee *et al*., 2022 [17]	78,380	64	65	-	34	78	-	-	-	-	-	-	57	58	-
Godoy *et al*., 2023 [9]	28,039	73	66	-	35	77	-	-	10	-	-	87	-	74	-

Abbreviation: BB, Beta-blocker; BMI, Body Mass Index; PCI, Percutaneous Coronary Intervention; CABG, Coronary Artery Bypass Grafting; ACEi/ARB, Angiotensin-Converting Enzyme inhibitor/Angiotensin Receptor Blocker; CCB, Calcium Channel Blocker.

**Fig. 2.**
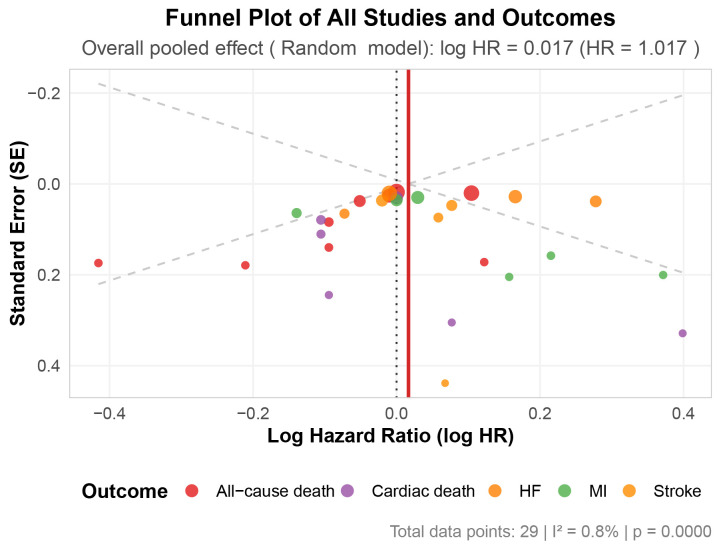
**Funnel plot**. Abbreviation: HF, heart failure; MI, myocardial infarction.

Publication bias was assessed using funnel plots and Egger’s regression test (Fig. 2). The funnel plot, which included 37 data points representing all cardiovascular outcomes across the included studies, appeared relatively symmetric around the overall pooled effect estimate (HR = 1.022; 95% CI: 0.982–1.062). Egger’s test showed no evidence of significant publication bias (*p* = 0.975). The overall heterogeneity across all outcomes was minimal (I^2^ = 0.8%, *p* < 0.001), indicating high consistency among the included studies. Additionally, Peter’s test showed no significant publication bias for cardiac death (*p* = 0.947), MI (*p* = 0.445), stroke (*p* = 0.659), and HF (*p* = 0.778), while possible publication bias was detected for all-cause death (*p* = 0.018) (**Supplementary Table 1**). Risk of bias assessment is presented in **Supplementary Table 2**.

### 3.2 Effects of Beta-blockers on Cardiovascular Outcomes

**Fig. 3.**
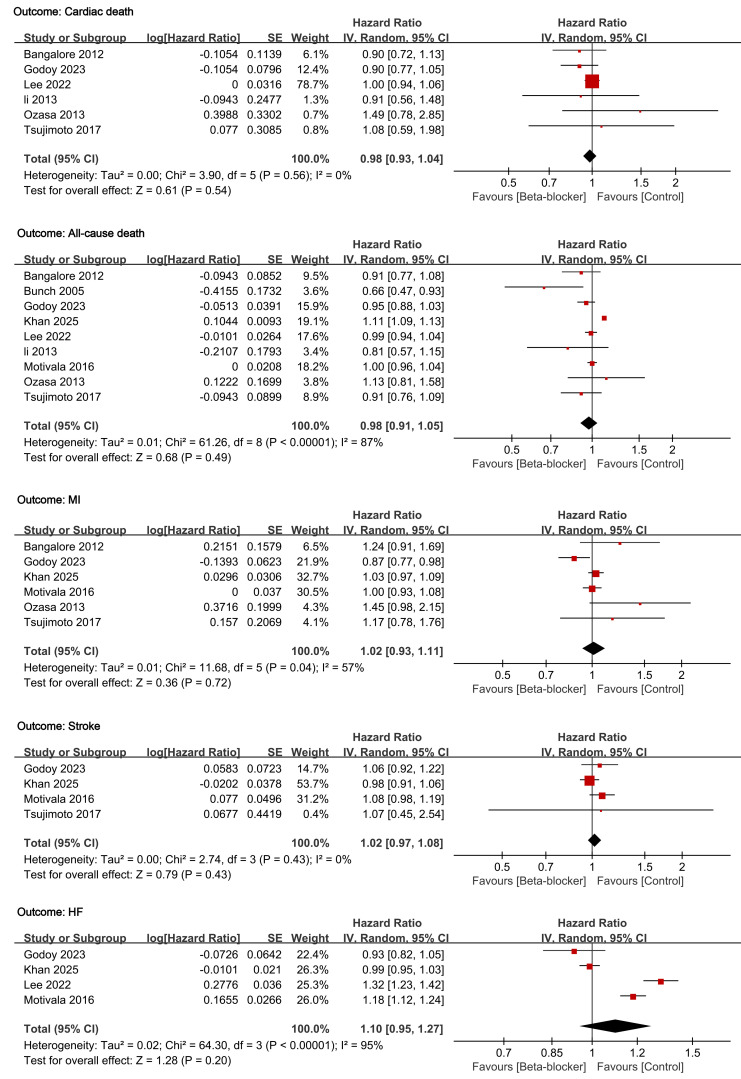
**Forest plots of the effects of beta-blockers on cardiovascular outcomes in patients with stable ischemic heart disease**.

The cardiovascular effects of beta-blocker therapy across five outcomes are presented in Fig. 3. Beta-blocker treatment demonstrated no significant benefit for the primary endpoint of cardiac death (HR 0.98; 95% CI: 0.93–1.04; *p* = 0.54). Secondary outcomes similarly demonstrated no significant effects: all-cause death (HR 0.98; 95% CI: 0.91–1.05; *p* = 0.49), MI (HR 1.02; 95% CI: 0.93–1.11; *p* = 0.72), stroke (HR 1.02; 95% CI: 0.97–1.08; *p* = 0.43), and HF (HR 1.10; 95% CI: 0.95–1.27; *p =* 0.20). All confidence intervals crossed unity, indicating no statistically significant effects. Heterogeneity analysis revealed substantial variation across outcomes. High heterogeneity was observed for all-cause death (I^2^ = 87%) and HF (I^2^ = 95%), while moderate heterogeneity was present for MI (I^2^ = 57%). Conversely, cardiac death and stroke demonstrated no heterogeneity (I^2^ = 0% for both).

### 3.3 Subgroup Analyses

**Fig. 4.**
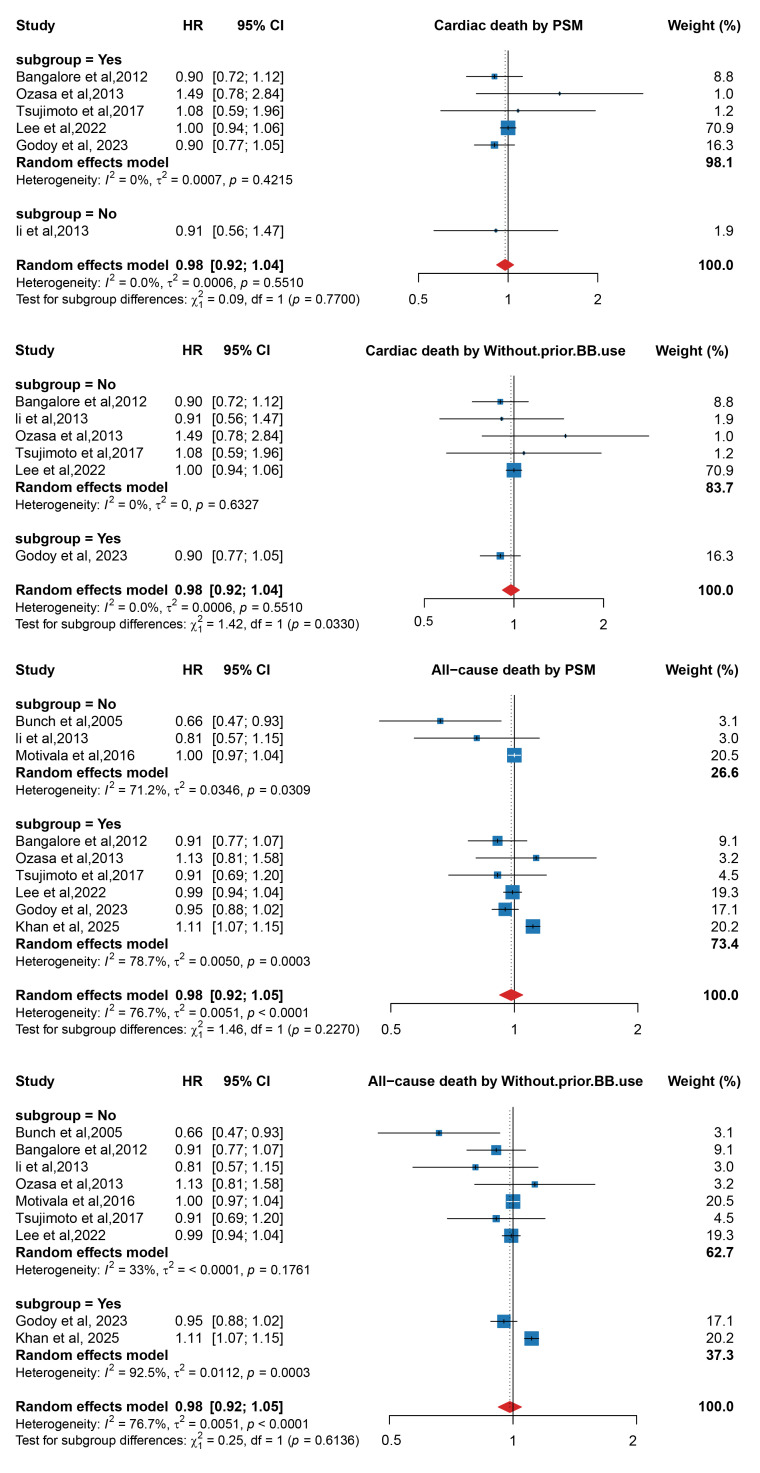
**Subgroup analyses of beta-blockers effects on all-cause death and MACE stratified by PSM status and prior beta-blocker use**. Abbreviation: BB, Beta-blocker; MACE, major adverse cardiovascular events; PSM, propensity score matching.

To explore potential sources of heterogeneity and identify patient populations who might derive differential benefits from beta-blocker therapy, we conducted subgroup analyses according to two predefined variables: propensity score matching status and explicit exclusion of patients with prior beta-blocker use (Fig. 4). For cardiac death, when stratified by propensity score matching status, no significant differences were observed between subgroups (*p* = 0.77). Studies employing propensity score matching showed neutral effects (HR = 0.98, 95% CI: 0.92–1.04), while studies using multivariate adjustment demonstrated similar neutral effects (HR = 0.91, 95% CI: 0.56–1.47). When stratified by prior beta-blocker use exclusion criteria, a statistically significant subgroup difference was detected (*p* = 0.03). Studies that did not explicitly exclude patients with prior beta-blocker use showed neutral effects (HR = 1.00, 95% CI: 0.94–1.06), whereas the single study that explicitly excluded prior users demonstrated a trend toward benefit, although not statistically significant (HR = 0.90, 95% CI: 0.77–1.05). For all-cause death, when stratified by propensity score matching status, no significant differences were observed between subgroups (*p* = 0.23). Studies without propensity score matching demonstrated a slight protective trend (HR = 0.85, 95% CI: 0.71–1.02), while studies with propensity score matching showed neutral effects (HR = 1.00, 95% CI: 0.94–1.07). When stratified by prior beta-blocker use exclusion criteria, no significant subgroup difference was found (*p* = 0.61). Studies that did not explicitly exclude patients with prior beta-blocker use showed neutral effects (HR = 0.99, 95% CI: 0.94–1.04), while studies that explicitly excluded prior users showed similar neutral effects (HR = 0.98, 95% CI: 0.91–1.05). Overall, subgroup analyses failed to identify specific patient populations that would clearly benefit from beta-blocker therapy.

## 4. Discussion

This systematic review and meta-analysis demonstrates that beta-blocker therapy was not significantly associated with cardiovascular endpoints in patients with stable CAD and preserved left ventricular function. These findings contribute to an evolving and contradictory body of evidence regarding beta-blocker efficacy in this population.

Current clinical practice guidelines have increasingly questioned the routine use of beta-blockers in stable CAD patients without prior MI or left ventricular dysfunction. The most recent 2023 AHA/ACC/ACCP/ASPC/NLA/PCNA Guideline for the Management of Patients with Chronic Coronary Disease made significant changes to beta-blocker recommendations, stating that “long-term beta-blocker therapy is not recommended to improve outcomes in patients with chronic coronary disease in the absence of MI in the past year, left ventricular ejection fraction ≤50%, or another primary indication for beta-blocker therapy” (Class 3: No Benefit) [19]. Similarly, the 2019 European Society of Cardiology Guidelines for the management of chronic coronary syndromes provide no specific Class I recommendation for beta-blockers in patients without HF or recent MI [20]. This uncertainty was reinforced by the 2021 meta-analysis by Arero *et al*. [8], which pooled six observational studies (n = 774,089) and found no significant reduction in MI or cardiovascular death with beta-blocker therapy. However, this consensus was challenged in 2023 when Godoy *et al*. [9] published a rigorously designed population-based cohort study in the Journal of the American College of Cardiology. Their study of 28,039 patients with angiographically confirmed stable CAD demonstrated a significant 8% relative risk reduction in major cardiovascular events (HR 0.92, 95% CI: 0.86–0.98, *p* = 0.006), primarily driven by reduced MI hospitalizations [9]. This positive finding reignited the debate about beta-blocker efficacy and highlighted the need for updated evidence synthesis.

Our comprehensive meta-analysis, incorporating all available evidence including the Godoy study, found no significant benefit for cardiac death (HR 0.98, 95% CI: 0.93–1.04, *p* = 0.539), suggesting that the overall evidence remains insufficient to support routine beta-blocker use in stable CAD patients. Although our subgroup analyses did not identify specific patient populations that consistently benefit from beta-blocker therapy, examining potential explanatory factors for the divergent results between studies may generate important hypotheses for future research. The divergent results from the Godoy study may stem from several key factors: First, their new-user design with a 1-year washout period specifically included only beta-blocker-naïve patients, designed to eliminate confounding from prior exposure and capture the true effects of beta-blocker initiation. However, our subgroup analysis of studies that explicitly excluded patients with prior beta-blocker use still failed to demonstrate significant benefits for cardiac death (*p* = 0.2330), suggesting that even in treatment-naïve populations, beta-blocker efficacy remains uncertain. Second, strict patient selection criteria created a unique study population—patients >66 years old with angiographically confirmed significant stenosis (>50% left main or >70% major vessels), ensuring anatomically high-risk features while excluding younger low-risk populations. This “triple-screening” strategy may have identified a specific population most likely to benefit from beta-blockers. Third, bisoprolol used by 66% of patients has higher β1 selectivity and longer half-life, potentially providing more stable cardioprotective effects [21]. Fourth, the optimized modern secondary prevention treatment background may be crucial, with statin use reaching 87% in the Godoy study, significantly higher than 52–72% in earlier studies. This suggests that beta-blocker benefits may only manifest in the context of comprehensive optimized therapy, explaining why beta-blockers alone show no benefit in suboptimal treatment settings but demonstrate benefit in modern optimized care. In contrast, the 2025 US study by Khan *et al*. [18], despite similarly employing a new-user design, found an 11% increase in all-cause mortality. Key differences include Khan’s study covering all age groups, higher chronic obstructive pulmonary disease proportion (34.3% vs 22.7%), and being based on electronic medical record data from a diversified healthcare system. These contrasting findings underscore the complexity of the relationship between beta-blockers and cardiovascular outcomes in stable CAD, highlighting the need for rigorous prospective studies to confirm which specific patient subgroups, if any, might benefit from this therapy.

Our meta-analysis revealed substantial heterogeneity, particularly for all-cause death (I^2^ = 87%) and HF (I^2^ = 95%). Beyond the factors discussed in our subgroup analyses and exploration of the Godoy study, several additional elements likely contributed to this variability. Geographic diversity across studies (spanning North America, East Asia, and international settings) introduced differences in healthcare systems and practice patterns. The specific beta-blockers used varied from predominantly cardioselective agents in some studies to more diverse pharmacological profiles in others. Background therapy levels also differed markedly, with variations in contemporary evidence-based therapies potentially creating different contexts for beta-blocker efficacy. Methodological differences in follow-up durations and approaches to confounding control further contributed to the observed heterogeneity. These multiple sources of clinical and methodological variability may help explain the absence of significant associations between beta-blocker therapy and cardiovascular outcomes in our analysis.

Despite our overall null findings, it would be premature to conclude that beta-blockers provide no benefit in stable CAD patients. The divergent results between studies, particularly the positive findings in Godoy’s carefully selected elderly population with high-risk anatomical features, suggest that patient heterogeneity may be the key factor determining treatment response. Rather than reflecting true inefficacy, our results may highlight the limitations of current research methodologies in identifying the optimal candidates for beta-blocker therapy. The challenge lies in developing robust clinical criteria to identify patients who would truly benefit from therapy. Future research should focus on establishing individualized treatment algorithms rather than pursuing blanket recommendations, with careful consideration of individual patient characteristics, comorbidities, and risk profiles.

This study has several important limitations to consider. First, all included studies were observational, and despite employing advanced confounding control methods, residual and unmeasured confounding cannot be completely excluded. Second, significant methodological heterogeneity existed between studies, including differences in exposure definitions, follow-up times, endpoint definitions, and statistical methods, potentially affecting result comparability. Third, we could not obtain individual patient data for more refined subgroup analyses, limiting precise identification of benefiting populations. Fourth, the relatively small number of included studies (n = 9) limits the robustness of publication bias assessment using funnel plots, although we supplemented this with Peter’s test to strengthen our evaluation. Fifth, most studies came from developed countries’ healthcare systems, potentially limiting global applicability to developing countries or different healthcare resource settings. Finally, rapid evolution of medical practice during observation periods, particularly introduction of novel antiplatelet agents and proprotein convertase subtilisin/kexin type 9 (PCSK9) inhibitors, may have affected assessment of beta-blockers’ relative therapeutic value.

In conclusion, we conducted a meta-analysis of 9 observational studies investigating the effects of beta-blocker therapy on cardiovascular endpoints in stable CAD patients. Our meta-analysis showed no significant association between beta-blockers and reduced cardiac death incidence in stable CAD patients. These findings provide additional contemporary evidence supporting current guideline recommendations from both AHA/ACC (Class III: No Benefit) and ESC regarding beta-blocker use in this population. Although Godoy *et al*.’s [9] study showed positive signals in specific subgroups, overall evidence suggests that routine use of beta-blockers in this population may require careful reconsideration. Clinicians should conduct risk-benefit assessments based on individual patient characteristics rather than adopting “one-size-fits-all” prescribing patterns. To clarify beta-blocker indications in this patient subset and identify truly benefiting populations, rigorously designed randomized controlled trials are needed to improve evidence quality, particularly prospective studies targeting elderly patients with high-risk anatomical features.

## 5. Conclusion

In this meta-analysis of contemporary studies, beta-blocker therapy did not significantly reduce the incidence of cardiovascular events in stable CAD patients with preserved left ventricular function without other indications for beta-blockers. These findings provide additional contemporary evidence supporting current guideline recommendations from both AHA/ACC and ESC regarding beta-blocker use in this population. Clinicians should conduct individualized risk-benefit assessments rather than adopting routine prescribing patterns.

## Availability of Data and Materials

The data used to support the results of this study are available from the corresponding author upon request.

## Supplementary Material

Supplementary material associated with this article can be found, in the online version, at https://doi.org/10.31083/RCM44520.
