# Supplementary material for: Beta-Blockers in Stable Coronary Artery Disease: A Systematic Review and Meta-Analysis of Observational Studies
Source: Rev Cardiovasc Med. 2025 Dec 23;26(12):44520. doi: 10.31083/RCM44520 (PMC12781013; doi:10.31083/RCM44520)
Supplement: Supplementary file 1 [file 2153-8174-26-12-44520-s1.zip › Supplementary Material.docx]

Supplementary Table 1. Peter's Test Results for Publication Bias Assessment

| Outcome | Statistic (*P*) | Bias Assessment |
| --- | --- | --- |
| Cardiac Death | -0.07 (0.947) | No significant bias |
| All-cause death | -3.09 (0.018） | Possible publication bias |
| MI | 0.85 (0.445) | No significant bias |
| Stroke | 0.51 (0.659) | No significant bias |
| HF | 0.32 (0.778) | No significant bias |

| Study | D1 | D2 | D3 | D4 | D5 | D6 | D7 | Overall |
| --- | --- | --- | --- | --- | --- | --- | --- | --- |
| Bunch 2005 | Moderate | Moderate | Low | Low | Moderate | Low | Low | Moderate |
| Bangalore 2012 | Low | Low | Low | Low | Low | Low | Low | Low |
| Ozasa 2013 | Moderate | Moderate | Low | Low | Low | Low | Low | Moderate |
| Li 2013 | Moderate | Low | Low | Low | Moderate | Low | Low | Moderate |
| Motivala 2016 | Moderate | Serious | Low | Low | Moderate | Low | Low | Serious |
| Tsujimoto 2017 | Low | Low | Low | Low | Low | Low | Low | Low |
| Lee 2022 | Low | Moderate | Low | Low | Moderate | Low | Low | Moderate |
| Godoy 2023 | Low | Low | Low | Low | Low | Moderate | Low | Moderate |
| Khan 2025 | Low | Low | Low | Low | Moderate | Low | Low | Moderate |

Supplementary Table 2. ROBINS-I Risk of Bias Assessment for Included Studies
